# Shaping farmers’ beliefs, risk perception and adaptation response through Construct Level Theory in the southwest Iran

**DOI:** 10.1038/s41598-023-32564-x

**Published:** 2023-04-10

**Authors:** Masoud Yazdanpanah, Tahereh Zobeidi, Laura A. Warner, Katharina Löhr, Alexa Lamm, Stefan Sieber

**Affiliations:** 1 Department of Agricultural Extension and Education, Agricultural Sciences and Natural Resources University of Khuzestan, Mollasani, Iran; 2 Cooperation and Transformative Group, International Institute for Applied Systems Analysis (IIASA), Laxenburg, Austria; 3 Department of Agricultural Education and Communication, Institute of Food and Agricultural Sciences, University of Florida, Gainesville, USA; 4 Leibniz Centre for Agricultural Landscape Research (ZALF), Muncheberg, Germany; 5 Urban Plant Ecophysiology, Humboldt-Universitat zu Berlin, Berlin, Germany; 6 Department of Agricultural Leadership, Education and Communication, College of Agricultural and Environmental Sciences, University of Georgia, Athens, USA; 7 Resource Economics, Humboldt-Universitat zu Berlin, Berlin, Germany

**Keywords:** Environmental social sciences, Human behaviour

## Abstract

Due to the severe effects of climate change on the agricultural sector, urgent action is required on the part of farmers and is, indeed, critical to reducing climate change impacts. However, reports globally revealed farmers’ engagement in climate change adaptation is still insufficient, ambivalent, and inconsistent and farmers do not consider adaptation to be urgent. Researchers have argued that this issue is rooted in psychological biases beside other factors. Therefore, the aim of this study is to evaluate how psychological distance determines climate change beliefs, risk perception and adaptation strategies among Iranian farmers. A cross-sectional paper-based survey was conducted in the *Dasht-e Azadegan* county of Khuzestan province in southwest Iran. The study sample consisted of 250 farmers selected through a multi-stage random sampling process. An expert panel review and a pilot study were conducted to confirm convergent validity and reliability of the scales. The results confirm that all four dimensions of psychological distance influence water management adaptation strategies and non-farm activities. Moreover, all psychological dimensions, except the temporal dimension, affect adaptation in farming management. Thus, making climate change more proximal to decision makers could be a strategic way of encouraging individuals to take adaptive actions. This study emphasizes that concepts of psychological distance can be applied to help organizations (e.g., agriculture extension services) to understand farmers’ risk perceptions and responses to climate change impacts and improve risk communication to better engage farmers in climate action.

## Introduction

Climate change is having, and will continue to have, extensive repercussions on agricultural productivity. Devastating impacts are foreseen on the agricultural sector with its many inherent vulnerabilities^1,2^, including a significant reduction in crop yield, food security and farmers' welfare (directly). This, in turn, could (indirectly) cause other crises—economic, social, environmental and even political—exacerbating poverty levels and making poverty even more widespread in rural populations^3,4^. These factors would increase the development challenges already faced by many developing countries^5–7^ and, in particular, undo decades of agricultural development efforts^8^.

Climate change is thus a social risk issue and has become a key consideration for public policy^9,10^. As some have argued, good development policy is good adaptation policy. It is thus imperative for farmers to adapt to climate change and reduce or avoid its adverse impacts so as to protect their livelihoods and food security, enhance the sustainability of their farms, and contribute to the resilience of the agriculture sector as a whole^11,12^. Farmers’ adaptation to climate change is essential for job retention, protection of local and global environments, and sustainable development^13–16^.

The need to adapt has led to increasing calls for initiatives at different levels of climate politics from local to international to help farmers and other stakeholders to produce adequate amounts of food and fiber while protecting the environment and natural resources^17,18^. Although adaptation requires the participation of multiple stakeholders at different levels, farmers in rural areas comprise the most important level as they are not only the critical decision-makers with respect to natural resource conservation, water management and agricultural practices but also the most severely affected by climate change^19^. Unless adaptation is implemented at the farm level, all other adaptation efforts will fail, including policy design and planning at national and international scales^20^. Urgent action is thus required on the part of farmers and is, indeed, critical to reducing climate change impacts^19,21^. Reports from around the world have revealed that despite farmers acknowledging the occurrence of climate change and even having concerns about it, their engagement in climate change adaptation is still insufficient, ambivalent, and inconsistent; action to reach set adaptation policies is lacking or farmers in both developed and developing countries do not consider adaptation to be urgent compared to other issues^22–24^. One explanation is that climate change may be perceived as a psychologically far-off (distal) concern compared to market variability and food security, which are immediate (proximal) concerns^25^.

For example, Tucker et al.^26^ found that farmers were far more concerned about market volatility than climate change. Tzemi and Breen^27^ also found that Irish farmers see climate change as only a long-term threat to their farms rather than a current source of risk. Wise et al.^28^ revealed that climate change was rarely the sole motivator for implementing adaptation behavior.

The lack of concern is due to ambiguity and uncertainty about climate change and adaptation^29^ and is rooted in psychological biases^30^. Climate change is often not perceived as a constant phenomenon but considered only during extreme events such as severe drought, floods or other weather fluctuations. In times when these extremes are absent, factors such as the market and food supply are the main drivers of behavior^31^. It is important to note that a bulk of studies^32–36^ argue that adaptation planners treat connected and very important risks as discrete risks as somehow disconnected from one another. Nhamo et al.^37^, showed that underdeveloped, lack of resources to adapt, poor institutional and legal frameworks affected adaptation. Chenani et al.^38^ confirm that social and cultural barriers, market and economic, technological, knowledge and informational and formal institutional barriers, are different categories of obstacles to adaptation. These studies declare that farmers face multiple stressors which can have an effect on the decision-making and response of farmers at the same time, therefore adaptation options need to address multiple stressors and address so-called underdevelopment to allow farmers to more directly address climate risk. In essence, the problem is as much structural as it is dependent on social psychological factors (here psychological distance) are just one small piece of the story. Researchers^9,10,39^ have argued that one reason for farmers’ lack of action in climate change adaptation is likely related to a phenomenon called *psychological distance (PD)*—a construct that refers to the extent to which an event or object is perceived as being far from or close to the self over a range of dimensions, including time, geography, and social distance^40^. This psychological distance (proximal or distal) is related to perceiving events or objects in a concrete or abstract position. Thus, if a person perceives an object or event as psychologically close, they tend to see it as being more concrete. On the other hand, if they perceive an object or event to be psychologically distant, they will see is as being more abstract^41^.

Researchers have argued that psychological distance constructs have theoretical implications for behaviors and attitudes^41^. These theoretical implications likely have implications for climate change, given that climate change is a relatively slow, cumulative, and invisible process. As climate change risk cannot be directly experienced, it is perceived as having low salience^42^. Climate change risk is 'buried' in familiar natural processes such as temperature change and weather fluctuations^43^. Since farmers will likely engage in pro-environmental behaviors when environmental issues are confirmable via actual observation^44^, psychological distance affects their perceived reality of the magnitude of climate change threats. If farmers understand climate change risks only at an abstract level, they will be unlikely to interpret them as personally or immediately relevant. Thus, a distal perception of climate change may decrease acceptance of its reality, could undermine any motivation to take adaptive actions to mitigate impacts, and could delay responses to the risks posed by climate change^10,42,45–47^.

Given the distal perception of climate change is an obstacle to adaptive behaviors, it is critical to change farmers' perceptions to foster more proactive adaptation behavior in order to mitigate climate change impacts on the farmers themselves and prevent more severe climate impacts. First, it is necessary to understand how psychological distance shapes climate adaptation in order to develop strategies for presenting climate change information in a way that makes it more psychologically proximal to farmers. In fact, in terms of managing the communication of risk in, for example, agricultural extension and advisory services, it is important to recognize the extent to which adaptation strategies are influenced by the way in which a phenomenon like climate change is perceived and considered by different stakeholders like farmers and other producers. Effective climate science communication with farmers and bidirectional engagement in adaptation and mitigation strategies are lacking^19,21^. This raises the question as to whether climate change risks can be made a more proximal issue by presenting them as more real, local, relevant, and immediate to farmers to encourage them to adopt coping and adaptation strategies^10^. Therefore, the aim of this study is to evaluate how psychological distance determines adaptations decisions in three different categories of common adaptation strategies in Khuzestan, including water management, farm management and non-agricultural activities in a case study of Khuzestan farmers in Iran. The research seeks to identify how psychological distance affects farmers' beliefs that climate change is anthropogenic as well as their risk perception and adaptation to climate change.

## Theoretical basis and research hypothesis

This study aims to examine impacts of psychological distance on farmers' belief that climate is anthropogenic, their perception of climate change risks and adaptation responses, using Construct Level Theory (CLT^48^) as a lens^9^. CLT describes the relationship between psychological distance and the extent to which people's perceptions of events and objects are concrete or abstract^48^. Based on psychological distance, CLT can explain how an object or concept is perceived as being familiar and concrete, or, on the contrary, abstract and distant from the mind.

The success of the CLT in predicting individual perceptions and behaviors to date has led to its extensive application in the a range of fields, including but not limited to economic/consumer psychology and behavioral economics^49,50^, pro-environmental attitudes and behaviors^51^, climate change mitigation and adaptation behaviors^52,53^, green purchasing intentions^54^, and consumer behavior^55^. Only a few studies have either partially^56^ or more fully tested CLT and PD within the realm of farmers and climate change^10,41^.

Most of the past studies in this realm undertaken in developed countries have focused primarily on the general public^57^ although other segments of society may warrant particular scrutiny^58^. For example, research investigating the links between CLT and farmers’ adoption of adaptation practices in climate change issues is noticeably lacking among the agriculture sector in developing countries. A better understanding of adaptation measures that farmers use and psychological distance is critically important in terms of informing organizations concerned with the communication of risk to farmers (e.g., agriculture extension services). Proximalizing climate change is an encouraging communication strategy for extension and advisory services in terms of promoting farmers' adoption of climate change adaptation practices. Also, a vital role of psychological distance is that it impacts what information individuals specifically notice when they consider (that is, construe) an object or event, and when they then make choices. In other words, CLT explains that growers perceiving climate variability as distant should be given an opportunity to interrelate this topic with other real and situation-specific information to regulate individual responses^59^. In essence, CLT can provide a platform for investigating situation-specific versus abstract and generalized visualization on the part of farmers in the area of climate-related judgments and behavioral intentions^59^.

CLT assumes that people are in a better position to envision and make decisions about events and objects when they are psychologically close (proximal) to them than when they are psychologically distant (distal)^9^. Spence et al.^10^ argued that CLT has the potential to reveal how connections between psychological distance and farmers' risk perceptions of climate change might be used to promote adaptation behavior. In this study we assume that exploring the relation between different aspects of psychological distance, beliefs, risk perception, and adaptation response can be significant for formulating more effective communication regarding the risks of climate change.

The results of this study also can contribute to the literature of psychological distance and climate change adaptation behaviours, given that the relationship between the two currently requires clarification due to conflicting results (see Schuldt et al.^47^). For example, Schuldt's (2018) study shows in an experimental design that those who judged climate change as geographically closer and more concrete did not have more political support. In fact, studies show that localizing climate change by itself is unlikely to increase engagement.

CLT delineates four core dimensions of psychological distance pertaining to the physical distance, time distance, uncertainty, and social acceptance an object/event—the geographic/spatial dimension (GD), the social dimension (SD), the temporal dimension (TD), and the hypothetical (of uncertain nature) dimension (HD)^60^, respectively. These dimensions, though having no commonalities^40^, are interrelated^30,41,61^. Individuals who perceive climate change as having greater psychological distance might think that climate change is a remote threat happening in distant places, impacting other people and regions that are geographically far away (GD). People who perceive climate change as having greater psychological distance may also assume that although climate change is happening, the impacts will be more severe for people in the future (TD)^10,62^.

**Figure 1:**
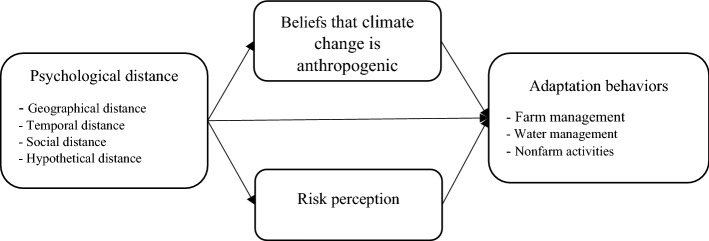
Theoretical framework based on CLT.

It is frequently assumed by researchers that most people perceive climate change as a distant phenomenon along all of the four dimensions^51^. Based on CLT, it is assumed that when people perceive climate change as being a psychologically distant construct, its threat and risk are less real, tangible, or relevant. Psychological distance could potentially hinder action^41^ and thus act as an important barrier to climate change adaptation^39,42,63–65^. Here, we investigate the relationship between all four naturally arising dimensions of psychological distance and farmers’ engagement in adaptation behavior. Figure 1 shows a theoretical framework to explain how psychological distance dimensions could affect beliefs, risk perception and adaptation practices including water management, farm management and non-farm management or activities. Based on the research framework, we propose the following hypotheses:

- GD (H1), TD (H2), SD (H3) and HD (H4) have negative direct effects on belief that climate change is anthropogenic
- GD (H5), TD (H6), SD (H7) and HD (H8) have negative direct effects on risk perception.
- Beliefs that climate change is anthropogenic has a positive direct effect on risk perception (H9).
- GD (H10), TD (H11), SD (H12) and HD (H13) have negative direct effects on adaptation–farm management.
- Beliefs about causes of climate change (H14) and risk perception (H15) have positive direct effect on adaptation-farm management.
- GD (H16), TD (H17), SD (H18) and HD (H19) have negative direct effects on adaptation-water management.
- Beliefs about causes of climate change (H20) and risk perception (H21) have positive a direct effect on adaptation-water management.
- GD (H22), TD (H23), SD (H24) and HD (H25) have negative direct effects on adaptation-nonfarm management.
- Beliefs that climate change is anthropogenic (H26) and risk perception (H27) have positive a direct effect on adaptation-nonfarm activities.

## Methodology

### Instrument and measure

The current study was conducted using a cross-sectional paper-based survey instrument. An extensive literature review of relevant previous studies was used in designing the questionnaire. The questionnaire (Appendix 1) consisted of two main parts: (1) research model variables and (2) demographic and socioeconomic variables. The details of the measurement scales and indicators adapted for measuring research model variables consisted of 17 items scaled to measure farmers’ psychological distance regarding climate change^40,66^. Five items were used to measure farmers’ beliefs^19^ and six items were used to measure farmers’ risk perception^67,68^. While researchers^69–71^ have categorized adaptation behavior in different ways, we selected three scales for measuring farmers’ adaptation behavior, including farm management (3 items), water management (3 items), and non-farm activities (5 items). A panel of experts in environmental psychology, climate sciences, and agricultural extension conducted an expert panel review to establish validity of the final statements. To investigate the reliability of the scales, the questionnaire was pretested with 30 farmers in a pilot study.

All questions were measure using a five-point Likert scale. For perception-oriented questions, respondents were asked to indicate their views on items with available responses ranging from strongly disagree (1) to strongly agree (5), and for the behavior questions, respondents were asked to indicate the extent to which they engaged in the adaptation strategies on their farm with responses ranging from very low (1) to very high (5).

### Case study region

**Figure 2:**
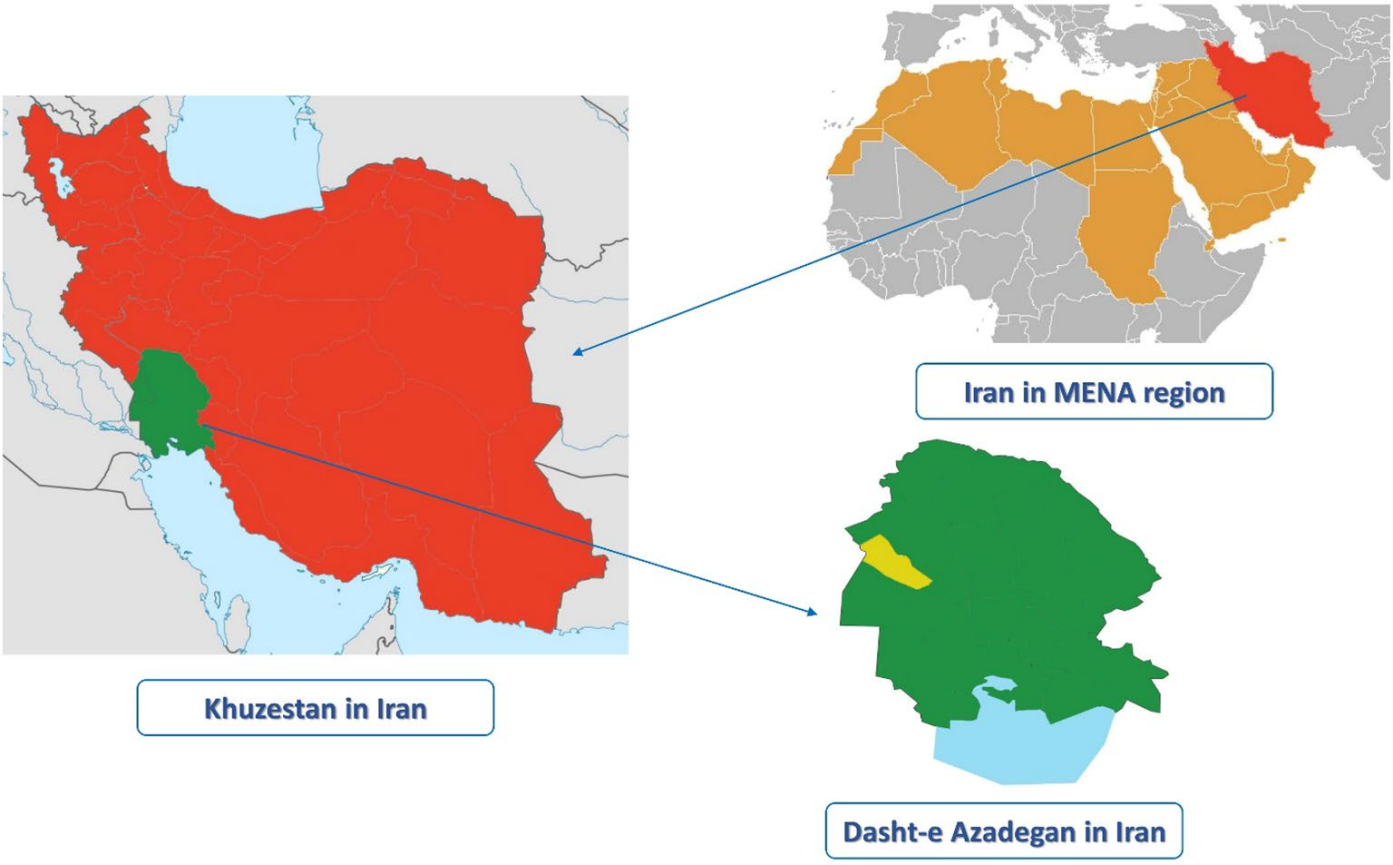
Location of case study region on map.

The current study was conducted in the *Dasht-e Azadegan* county of Khuzestan province in southwest Iran (Fig. 2). Susangerd is the main and central city in the Dasht-e Azadegan county. At the 2006 census, the county's population was 126,865 in 22,021 households. The latitude of Dasht-e Azadegan is 31.55806, and the longitude is 48.18083.

The area has a history of recurrent drought and dust storms. Khuzestan province is one of the most important for agriculture and livestock production in Iran. It is a fertile plain through which many large Iranian rivers flow. This province is the largest producer of cereals (wheat, barley, corn, and rice) and sugarcane, one of Iran's strategic products. It is also the largest producer of vegetables such as out-of-season leafy vegetables, onions, garlic, eggplant, tomatoes, squash in Iran. In addition, the province is an important producer of citrus and dates. According vast studies in Khuzestan^20,72,73^ the region’s farmers have observed impacts of climate change including lower productivity on farm due to disease and pests, desertification and decreasing soil fertility.

### Data collection

The study sample consisted of 250 farmers from *Dasht-e Azadegan* county of Khuzestan province in southwest Iran selected through a multi-stage random sampling process. Based on Krejcie and Morgan^74^, 250 farmers were selected as the sample size. Attempt were made to draw a sample representative of Dasht-e Azadegan’s socio-demographic characteristic with random sampling used to target an unbiased representation of the larger population. The primary sampling units were subdivided to two districts (Bakhsh) of the Central district and Bostan district. The sample size was indicated according to the population of farmers in each district proportional to the population of the whole district. Finally, farmers were randomly selected from each village in the district.

Research data were gathered through interviews and completion of the structured questionnaire in June–September 2019. The average time taken by each interviewee to complete the questionnaire was 35 min. Interviews were conducted in a place that was comfortable for the farmers. Participation in the study was voluntary. Before the interview, the purpose of the interview and related research was explained and farmers were assured that the data given were confidential and anonymous. Data gathering was carried out by an expert from the region to facilitate communication with farmers due to his familiarity with the language and customs.

All procedures performed in studies involving human participants were in accordance with the ethical standards of the institutional and/or national research committee and with the 1964 Helsinki declaration and its later amendments or comparable ethical standards. All materials and methods are performed in accordance with the instructions and regulations and this research has been approved by a committee at Agricultural Sciences and Natural University of Khuzestan, Iran. Informed consent was obtained from all subjects.

### Data analysis

Assessment of the research model was performed using the PLS-SEM technique and SmartPLS software (Version 3.2.8) PLS-SEM is primarily used to create explanatory models^75^. PLS-SEM follows a combination-based approach to structural equation modeling in which the algorithm uses a weighted combinations of indices to statistically model latent variables^76^. The internal consistency of the scale was tested via factor loading (FL) and composite reliability (CR). Such indicators should be larger than 0.7 to represent a high level of reliability of the internal consistency. The indicator of average variances extracted (AVE) was used to examine convergent validity. The acceptable threshold of AVE is 0.5 or higher^77^. AVE numbers and latent variable correlations were evaluated to check discriminant validity or divergent validity. The square root of AVE of each latent variable should be greater than the correlation of that variable with all other variables^78^. Currently, the only approximate model fit criterion implemented for PLS path modeling is the standardized root mean square residual (SRMR)^79^ which was equal to 0.07 in the current study.

## Results

### Socioeconomic characterization of the respondents

Farmers' ages ranged from 24 to 73, with a mean age of 44.68 years (SD = 10.85) The mean education level of farmers was 7.15 years (SD = 4.72). Education level ranged from no formal education to 18 years' education. The average work experience in agriculture jobs was 15.94 years (SD = 9.99). The minimum agricultural experience was 2 years and the maximum was 55 years. Of the farmers, 248 (99.20%) were male and 2 (0.80%) were female.

### Structural equation modeling results

#### Evaluation of measurement model

As shown in Table 1, the standardized factor loading of all analysis items for the relevant constructs was above the acceptable threshold of 0.5 and statistically significant at the 0.01 level.

**Table 1:** Evaluation of measurement model.

Constructs, validity and reliability	Item	Factor loading	T
Geographic distance (GD) AVE: 0.545, CR: 0.802, α: 0.701	GD1	0.684	6.303
	GD2	0.562	2.819
	GD3	0.834	18.255
	GD4	0.822	24.215
Temporal distance (TD) AVE: 0.588, CR: 0.830, α:0.718	TD1	0.652	9.305
	TD2	0.886	34.717
	TD3	0.890	40.671
	TD4	0.587	5.499
Social distance (SD) AVE: 0.645, CR: 0.878, α: 0.814	SD1	0.823	20.179
	SD2	0.888	40.407
	SD3	0.735	16.294
	SD4	0.756	16.387
Hypothetical distance (HD) AVE: 0.575, CR: 0.871, α: 0.816	HD1	0.749	12.736
	HD2	0.746	15.420
	HD3	0.801	24.507
	HD4	0.742	11.370
	HD5	0.751	15.115
Belief (BE) AVE: 0.557, CR: 0.858, α: 0.804	BE1	0.922	73.501
	BE2	0.587	7.978
	BE3	0.647	10.786
	BE4	0.617	6.885
	BE5	0.890	58.900
Risk perception (RP) AVE: 0.583, CR: 0.893, α: 0.858	RP1	0.700	12.898
	RP2	0.717	15.376
	RP3	0.826	17.689
	RP4	0.848	24.272
	RP5	0.684	7.961
	RP6	0.792	13.988
Farm management (FM) AVE: 0.601, CR: 0.818, α: 0.701	FM1	0.829	18.152
	FM2	0.579	5.701
	FM3	0.883	39.543
Water management (WM) AVE: 0.667, CR: 0.857, α: 0.748	WC1	0.916	69.441
	WC2	0.770	12.188
	WC3	0.755	18.254
Non-farm activities (NFA) AVE: 0.533, CR: 0.849, α: 0.775	NFA1	0.868	28.649
	NFA2	0.600	7.222
	NFA3	0.735	17.827
	NFA4	0.767	12.085
	NFA5	0.650	10.281

Cronbach's alpha coefficient and the CR values for all latent constructs were higher than 0.7, showing the items of each construct to be internally correlated. The AVE values associated with all constructs were higher than 0.5, showing that all constructs had acceptable validity and reliability. The square root of the AVE of each of the constructs was greater than the correlation of that construct with other constructs (Table 2). The discriminant validity of the constructs in the proposed research model was thus confirmed. This means that the latent constructs were not highly correlated with each other.

**Table 2:** Correlations with square roots of the AVE.

Constructs	1	2	3	4	5	6	7	8	9
1-BE	0.766^**a**^								
2-FM	0.576**	0.875^a^							
3-GD	− 0.540**	− 0.828**	0.774^a^						
4-HD	0.623**	0.856**	− 0.665**	0.858^a^					
5-NFA	0.750**	0.806**	− 0.708**	0.821**	0.830^a^				
6-RP	0.570**	0.566**	− 0.442**	0.587**	0.756**	0.764^a^			
7-SD	0.470**	0.506**	− 0.471**	0.582**	0.771**	0.474**	0.803^a^		
8-TD	0.498**	0.756**	− 0.625**	0.641**	0.818**	0.625**	0.705**	0.750^a^	
9-WM	0.603**	0.738**	− 0.675**	0.688**	0.827**	0.580**	0.564**	0.570**	0.817^a^

^a^The square roots of AVE.

**Correlation is significant at the < 0.01 level.

#### Test of research hypotheses

The results showed that the research variables could predict 0.454, 0.533, 0.601, 0.645 and 0.667% of the variance of the variables of belief in climate change, risk perception, farm management, water management, and non-farm employment, respectively (Table 3). GD and HD had a significant effect on the belief in the occurrence of climate change, while TD and SD did not have a significant influence. TD and HD had a significant effect on risk perception. Belief in climate change also affected perceptions of risk.

**Table 3:** Results of research structural models.

Hypothesis	ƛ	T	Result	R^2^
H1: GD → BE	− 0.195	2.384	Confirm	0.454
H2: TD → BE	0.108	1.072	Reject	
H3: SD → BE	0.013	0.090	Reject	
H4: HD → BE	0.433	5.062	Confirm	
H5: GD → RP	− 0.118	1.019	Reject	0.533
H6: TD → RP	0.424	3.302	Confirm	
H7: SD → RP	0.016	0.159	Reject	
H8: HD → RP	0.220	2.269	Confirm	
H9: BE → RP	0.229	3.088	Confirm	
H10: GD → FM	− 0.388	5.183	Confirm	0.601
H11: TD → FM	0.347	6.012	Confirm	
H12: SD → FM	0.210	4.163	Confirm	
H13: HD → FM	0.539	9.753	Confirm	
H14: BE → FM	0.052	1.033	Reject	
H15: RP → FM	0.010	0.210	Reject	
H16: GD → WM	− 0.353	3.598	Confirm	0.645
H17: TD → WM	0.136	1.292	Reject	
H18: SD → WM	0.218	2.620	Confirm	
H19: HD → WM	0.207	2.137	Confirm	
H20: BE → WM	0.149	2.029	Confirm	
H21: RP → WM	0.200	1.989	Confirm	
H22: GD → NFA	− 0.118	3.202	Confirm	0.667
H23: TD → NFA	0.155	3.502	Confirm	
H24: SD → NFA	0.287	7.384	Confirm	
H25: HD → NFA	0.191	4.944	Confirm	
H26: BE → NFA	0.241	6.152	Confirm	
H27: RP → NFA	0.230	4.964	Confirm	

All constructs related to psychological distance affected farm management; however, belief in climate change and perception of risk did not affect farm management. Moreover, all determinants of water management except TD had a significant impact on water management. All determinants of non-farm activities, including all dimensions of psychological distance, belief in climate change and risk perception, also had a significant impact on non-farm activities.

## Discussion

Warnings abound that climate change is an unprecedented and complex global hazard with potentially severe impacts on the agriculture sector and that to lessen those impacts, urgent adaptive response is required. However, farmers’ engagement still lags, as demonstrated by the body of literature stating that farmers deny the reality of the issue^18^. However, studies in Khuzestan, Iran such as Yazdanpanah et al.^20^ and Zobeidi et al.^68^ have shown that general beliefs about climate change were above average.

CLT suggests that an individual is better at anticipating and making decisions about events that are psychologically closer to them compared to those that are more psychologically distant^9^. While CLT proposes the critical role of psychological distance in influencing behavior^66^, it cannot predict that proximalizing climate change will automatically lead people to engage in adaptation behaviors (see Brügger et al.^59^; Schuldt et al.^47^; Loy and Spence^56^). CLT does not, in fact, clearly specify whether changing people's psychological distance can influence their cognitions and behaviors^47,56^. Researchers have demonstrated that psychological distance can provide a framework to explain beliefs in risk perception, willingness to act, and response to risk^39,80^. Thus it could reduce psychological distance by making climate change more familiar and relevant to farmers and consequently promote their action. In particular, psychological distance is a major challenge for risk communication organizations, including agriculture extension services wishing to encourage farmers to engage with climate change issues. These organizations aim to portray climate change in a way that brings the problem closer and make it more relevant to people and thus inspire political action^81^. Helping farmers to develop perceptions that climate change is closer or more proximal may be a strategy for encouraging individuals to take action^59^. The aim of this study was to investigate the impacts of psychological distance on farmers' belief in risk perception and adaptation responses. We investigated how perceptions that climate change is occurring proximally can influence farmers to adopt adaptive behaviors in water and farm management and non-farm activities.

Our results revealed that GD and HD significantly predict farmers’ belief that climate change is anthropogenic, while TD and SD have no impacts on farmers’ beliefs. In contrast to our finding, Kim and Ahn^82^ found that TD has a significant effect on students' attitude toward climate change mitigation behavior. Milfont et al.^83^ observed that GD has an effect on belief in climate change.

This finding revealed that variation in GD and HD influences Iranian farmers' beliefs. It is probable that farmers are certain about climate change occurrence in their region, given the recent recurrent and severe drought and other related hazards. GD and HD distances are related to physical distance from the effects of climate change and doubt and uncertainty as to its occurrence. As a result, farmers who think that the effects of climate change on other regions and countries are greater than where they personally live are less likely to believe in climate change. Farmers also believe that there is no consensus among scientists on climate change and its severity, or that scientists exaggerate the effects of climate change, which indicates greater HD which can reduce farmers' beliefs in climate change.

Furthermore, TD, HD, and farmers’ beliefs can positively predict farmers’ risk perception regarding the impact of climate change, while GD and SD have no impacts on farmers’ risk perception. Farmers who tend to perceive that climate change will have serious effects in the distant future probably have lower risk perception. Similarly, those whose perception of climate change is uncertain would have a lower perception of risk. The low level of concern is probably a result of a lack of immediacy^84^, meaning these individuals believe the effects of climate change will manifest themselves in the distant future and there is no need to act now. In partial contrast to our findings, Singh et al.^57^ found that only the TD of the four dimensions of psychological distance has a direct impact on individual concern about climate change. Furthermore, Jones et al.^39^ found that all psychological distance dimensions are significantly associated with climate change concern and mitigation intentions. Carmi and Kimhi^53^ found that environmental threats were perceived as psychologically distant and that this distance strongly affected the perceived severity of these threats and willingness to engage in adaptive behavior. Spence et al.^10^ found that there is a significant correlation between all dimensions of psychological distance, concerns about climate change, and intentions to perform sustainable behavior. They also showed that, in general, the closer the psychological distance, the higher the levels of concern. Perceived impacts on developing countries, as an indicator of SD, are also significantly associated with preparing for action on climate change.

According to our results, four dimensions of psychological distance can significantly predict farm management adaptation strategies including changing crop operations (sowing, planting, and harvesting), cultivation diversity and conservation tillage. However, farmers who think that climate change is exaggerated, and that it affects other people more than farmers, such as people who are disconnected from nature, will have relatively more farm management behavior and have engaged in more adaptation strategies (e.g., conservation tilling, diversifying cultivation and changing agricultural activities). Why this happened is probably because geographic distance is a more objective reflection of the reality of climate change. But when a person thinks about the social, hypothetical and temporal distance, s/he probably thinks that her/his distance is decreasing, and in fact, these distances are not constant, and the person sees himself in danger, so adaptation measures are inevitable for him. While neither farmers’ beliefs or risk perception have a significant effect on farm management, all dimensions of psychological distance and farmers’ beliefs and risk perception, except TD, significantly predicted water management adaptation strategies. Interestingly, all dimensions of psychological distance and farmers’ beliefs and risk perception can significantly predict non-farm adaptation strategies. In farm management strategies and non-farm activities, like farm management strategies, the effect of GD has been negative and the effect of other distances has been positive (if the effect was significant). Therefore, the direction of influence of psychological distance dimensions on all types of adaptation strategy has been similar.

Brügger et al.^59^ shed some light on this problem, by examining the extent to which proximal and distal risk perceptions of climate risk predict different types of adaptation behaviors. Singh et al.^57^ also found that TD does not influence support of adaptation policies. In contrast with our findings, Chen^51^ found that no significant correlation between psychological distance and pro-environmental behavior intentions. According to our results, belief that climate change is anthropogenic and people’s concern about it only affects water management and non-farm activities rather than farm management. This problem can be explained by farm management strategies being considered less effective in reducing risks, while water management strategies are responsive to the region's severe water shortage and non-farm strategies such as employment in services jobs resulting from the low incomes of farmers. In addition, the farm management strategies examined in this research mainly have lower income generation/profitability than employment in non-agricultural activities and water management.

## Conclusions and policy implications

The perception of psychological distance influences the belief of farmers in Iran that climate change is a human-caused risk and their adoption of a variety of adaptation measures. Such results are encouraging; they suggest that the inevitable reduction in perceived psychological distance to climate change will, in turn, lead to higher perceived risk, and more climate-positive attitudes and behaviors^41^. A policy strategy for communicators and policy makers is to reduce the perceived distance to climate change to the extent possible^41^. While there are risks associated with presenting climate change as being too near or too threatening, if people are unaware of the proximal occurrence of climate change, they will not take action. There is a fine line between a person's awareness of climate change and their concerns about it; however, the two are not so well integrated that a person believes that any response they perform can be effective. Individuals should be aware of the boundary between the two. Raising concerns through various programs should thus be undertaken with caution and should and be framed as solution-oriented. If the local impacts of climate change and the benefits of adapting to climate change are understood, people will be more inclined to take sustainable action. Here, our findings imply understanding the risks of climate change has led farmers to improve their water management and non-farm activities. Therefore, social media, including agricultural documentary programs, should warn about the potential or actual damages of climate change using concrete and proximal information. For example, farmers will likely understand the salience of expected impacts such as decreased quantity and quality of agricultural products, increases to the cost of living, or damages to the health sector locally.

The notion of certainty about the existence of climate change, namely the perception of a hypothetically short distance, can be undermined by skepticism about climate change in the media, which appears to reduce beliefs and concerns about it. Therefore, the media should be educated and provided with the tools to inform people about climate change using, for example, realistic statistics of changes in precipitation and temperature, as well as informative movies and documentaries. Agricultural extension and public campaigns can raise awareness of climate change and its constant impacts on farmers’/people’s living conditions in order to enhance perceived proximity to farmers and encourage them to engage more in active adaptation measures. Agricultural extension educators and risk communication should try to reduce the psychological distance and highlight the salience of its risks to help encourage adoption of adaptive behaviors.

Extension agents can improve farmers’ beliefs in climate change being human-made by reducing their GD and HD. In this way, documenting the localized climate change in the region, inviting specialists, comparing the past and present situation regarding the signs of climate change, and also showing the efforts of advanced countries in combating climate change will significantly help to increase farmers’ understanding. Considering the widespread use of social media among the farmers of the region, these media can be effective in increasing the belief of farmers about causes of climate change and its risk.

Both HD and TD have significant impact on risk perception. The agricultural extension organization can effectively shorten these two types of psychological distance and increase farmers' risk perception and thus increase their adaptive behavior. This type of action could be applied to different scenarios in order to enhance risk perception and reduce the psychological distance. In practice, different departments in the agricultural extension organization can be developed to focus on the psychological dimensions and human–environment interactions. Practitioners in these department can use real statistical data on climate change to directly update knowledge as they communicate and deliver programs among farmers and other stakeholders. In this regard, explaining and visualizing the signs of climate change in the region in different ways, describing the experience of the affected farmers, showing the weather events of the neighboring areas to the farmers will eliminate or reduce these two types of distance between the countries. Certainly, it will directly increase their understanding of risks related to climate change.

The findings of this study provide powerful empirical evidence that individuals' actions regarding climate change management and adaptation are affected by: (i) the extent to which they perceive climate change to be serious (risk perception), and (ii) whether they perceive the consequences of climate change to be proximal or distal temporally, geographically, social, and hypothetically. These dimensions of psychological distance affect almost all adaptation responses. However, our findings suggest that the TD—whether climate impacts occur now or in the future—is not a strong predictor of water management. This comes back to an important issue, that is, the type of water management methods which were measured in this research. It is natural that TD does not affect rainwater collection, which is a temporary adaptation strategy. In addition, although modern irrigation is considered a sustainable method of adaptation, it is relatively expensive, and therefore understanding the existence of a time gap with climate changes can justify the delay in the implementation of modern irrigation systems. In general, the results highlight the notion that making climate change appear closer to people is likely to be universally beneficial. In other words, making climate change more realistic, more local, more relevant, and more immediate may reduce alienation and denial and help people respond appropriately. Therefore, this study emphasizes the concept of public participation or stakeholder involvement. The participation of the key stakeholder group comprised of farmers is necessary to achieve sustainable adaptation. As other studies emphasize, a public participation and stakeholder engagement approach helps to facilitate the planning of ecosystem based adaptation^85^ and/or and/or sustainable adaptation^86^. Stakeholder engagement approaches may diverge based upon on the type of relationship where the stakeholders can provide information. Such engagement entails assessing vulnerability for climate adaptation, stakeholders’ contribution in the procedure of climate adaptation, assessing the existing and potential climate risk, evaluating the present and varying socio-economic circumstances, evaluating and refining the capability of adapting to, including adaptation policy and continuing the current adaptation procedure^86^.

Like other studies, this study is not without limitations. Using Construct Level Theory, we focus on only four dimensions of psychological distance, including geographical/spatial, social, temporal, and hypothetical/uncertainty. Although there are other dimensions to psychological distance, these four dimensions are at the core of most psychological distance discussions. Given this focus, we did not review the literature on related, but distinct, constructs that do not take into account the dimensions of psychological distance (such as attachment to place and spatial identity). It is suggested that these variables be examined in future studies to determine a higher percentage of variance in behavioral change. Moreover, climate change is an abstract issue. Experiencing impacts related to climate change can—at least in terms of HD—bring climate change from the abstract to the concrete. It is thus suggested that this impact be investigated in future studies.

Furthermore, it should be mentioned that one of the aspects of the distance issue regarding climate change is its effect on future generations, which was not investigated in this research and should be taken into consideration in future research.

This study also exhibits a gender bias. Therefore, it is suggested that future studies investigate the effect of psychological distance dimensions on beliefs and adaptation methods of women. It is also suggested to use multi-group analysis techniques to compare groups of women and men. Identifying the differences is likely to help design more targeted campaigns and programs.

## Supplementary Information

Supplementary Information.

## Data Availability

Some or all data, models, or code that support the findings of this study are available from the corresponding author upon reasonable request.
