# Supplementary material for: Shaping farmers’ beliefs, risk perception and adaptation response through Construct Level Theory in the southwest Iran
Source: Sci Rep. 2023 Apr 10;13:5811. doi: 10.1038/s41598-023-32564-x (PMC10085993; doi:10.1038/s41598-023-32564-x)
Supplement: Supplementary file 1 — Supplementary Information. [file 41598_2023_32564_MOESM1_ESM.docx]

Appendix 1

Table A1. Survey items and sources

| **Items** |
| --- |
| Risk perception (6 items) |
| I believe that, due to drought and climate change, my crop yields are declining. |
| Warming and climate change are reducing the yield of cereals (wheat, barley, corn, rice). |
| I believe that drought and climate change have a negative impact on the assets and income sources of farmers. |
| I believe that the cost of living will increase due to global warming and climate change. |
| In my opinion, the rate of diseases has increased due to climate change. |
| In my opinion, global warming has a great impact on human health. |
| Belief that climate change is anthropogenic (5 items) |
| In my opinion, climate change is caused mostly by human not natural factors. |
| In my opinion, climate change is caused by human activities. |
| In my opinion, industries and manufacturing are main causes of climate change. |
| In my opinion, cars and their emissions are the cause of climate change. |
| I am sure climate change is a phenomenon caused by humans. |
| Geographic / spatial distance (4 items) |
| When I think about the effects of climate change, I think of countries far away. |
| I think distant countries are feeling the effects more than people where I live. |
| Climate change is likely to affect distant countries. |
| My first thoughts on climate change are about its impact on my country. (Reverse coded) |
| Temporal distance (4 items) |
| Climate change is now hurting people all over the world. |
| Climate change is an immediate threat to people right now. (Reverse coded) |
| Future generations are more likely to feel the effects of climate change. |
| I doubt I will personally experience climate change in my lifetime. |
| Social distance (4 items) |
| The greatest effects of climate change are experienced by people like me. (Reverse coded) |
| Climate change is probably having a big impact on farmers like me. (Reverse coded) |
| Climate change is definitely affecting me and my family. (Reverse coded) |
| I do not think that climate change will have a significant effect on the farmers I know. |
| Uncertainty- hypothetical distance (5 items) |
| Most scientists do not agree climate change exists. |
| The severity of climate change has been greatly exaggerated. |
| I am sure that climate change is really happening. (Reverse coded) |
| I doubt the effects of climate change. |
| Due to changes in cooling and warming it is difficult to assess climate change and its effects. |
| To what extent do you do the following behaviors in the face of climate change?  Farm management (3 items) |
| Changing crop operations (sowing, planting, and harvesting) |
| Cultivation diversity |
| Conservation tillage |
| To what extent do you do the following behaviors in the face of climate change?  Water management (3 items) |
| Water conservation-saving |
| Modern irrigation |
| Rainwater collection |
| To what extent do you do the following behaviors in the face of climate change?  Nonfarm activities (5 items) |
| Employment in service jobs |
| Migration |
| Agricultural insurance |
| Reduce household expenses |
| Getting a loan |

*All research scales were measure using a five-point Likert scale.*
